# Evaluation of Intracochlear Trauma Caused by Insertion of Cochlear Implant Electrode Arrays through Different Quadrants of the Round Window

**DOI:** 10.1155/2015/236364

**Published:** 2015-07-05

**Authors:** Graziela de Souza Queiroz Martins, Rubens Vuono Brito Neto, Robinson Koji Tsuji, Eloisa Maria Mello Santiago Gebrim, Ricardo Ferreira Bento

**Affiliations:** ^1^Department of Medicine, Faculdade de Medicina, University of Sao Paulo, Avenida Dr. Eneás de Carvalho Aguiar 255, Sala 6167, 05403-000 São Paulo, SP, Brazil; ^2^Department of Otorhinolaryngology, Clinical Hospital, FMUSP, Avenida Dr. Eneás de Carvalho Aguiar 255, Sala 6167, 05403-000 São Paulo, SP, Brazil; ^3^Department of Radiology, Clinical Hospital, FMUSP, Avenida Dr. Eneás de Carvalho Aguiar 255, Sala 6167, 05403-000 São Paulo, SP, Brazil

## Abstract

*Hypothesis*. This study aimed to evaluate whether there is a difference in the degree of intracochlear trauma when the cochlear implant electrode arrays is inserted through different quadrants of the round window membrane. *Background*. The benefits of residual hearing preservation in cochlear implant recipients have promoted the development of atraumatic surgeries. Minimal trauma during electrode insertion is crucial for residual hearing preservation. *Methods*. In total, 25 fresh human temporal bones were subjected to mastoidectomy and posterior tympanotomy. The cochlear implant electrode array was inserted through the anterosuperior quadrant of the round window membrane in 50% of the bones and through the anteroinferior quadrant in the remaining 50%. The temporal bones were dehydrated, embedded in epoxy, serially polished, stained, viewed through a stereomicroscope, and photographed with the electrode arrays *in situ*. The resulting images were analyzed for signs of intracochlear trauma. *Results*. Histological examinations revealed varying degrees of damage to the intracochlear structures, although the incidence and severity of intracochlear trauma were not influenced by the quadrant of insertion. *Conclusions*. The incidence and severity of intracochlear trauma were similar in all samples, irrespective of electrode array insertion through the anterosuperior or anteroinferior quadrant of the round window membrane.

## 1. Introduction

Cochlear implants (CIs) represent a well-established treatment for severe and profound bilateral hearing loss. The development of CIs in the last 30 years is considered one of the milestones of modern medicine, and, to date, the outcomes of CIs have been remarkable and superior to those of any other type of neural prosthesis [1]. These results have encouraged the expansion of the selection criteria for CIs [2]. Therefore, the number of candidates with significant residual hearing who are eligible to receive CIs has increased, fostering several studies on the preservation of postoperative residual hearing in these patients. Intracochlear trauma during CI-related surgical interventions is one of the factors associated with residual hearing loss [3–5]. Previous studies have highlighted the possibility of electrode array insertion using atraumatic surgical techniques, which have been designated as soft surgeries [4, 6–9].

Among the steps involved in soft surgeries, electrode array insertion is the most frequently studied. CI arrays can be inserted via cochleostomy or through the round window (RW). According to Banfai [10], RW was the first choice of route for CI electrode array insertion. However, with the development of longer, thicker, and less flexible electrodes, insertion through RW became difficult and necessitated cochleostomy. Over the last few years, the development of thinner and more flexible electrodes has again enabled insertion through RW [11]. The possibility of electrode array insertion via these two distinct routes stimulated further comparative studies [12–15]. Numerous studies on cochleostomy have been conducted to determine any variations in intracochlear trauma according to its location. However, to date, no studies have evaluated differences in the degree of intracochlear trauma caused by electrode array insertion through different quadrants of the RW membrane.

Therefore, this study was conducted to determine differences in intracochlear trauma caused by CI electrode array insertion through the anterosuperior and anteroinferior quadrants of the RW membrane.

## 2. Materials and Methods

The Research Ethics Committee of the Department of Medicine, Universidade de São Paulo, approved this study. Twenty-five human temporal bones were retrieved within 24 h after death and were frozen and stored. To avoid divergence in relation to the laterality of the ears, the samples were first divided into two groups corresponding to the right and left ears. Then, they were randomly assigned to group 1, wherein the electrode array was inserted through the anterosuperior quadrant of the RW membrane, or group 2, wherein the electrode array was inserted through the anteroinferior quadrant.

**Figure 1:**
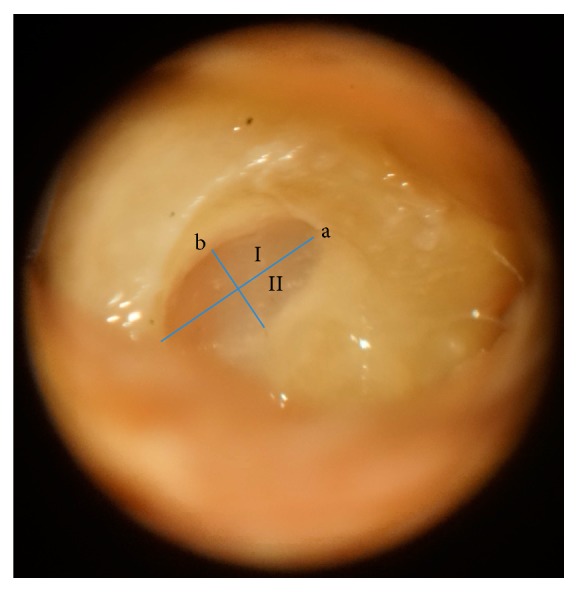
The round window membrane visualized through posterior tympanotomy after the removal of bony projections. Lines a and b dividing the quadrants of the round window membrane. I: anterosuperior quadrant. II: anteroinferior quadrant.

On the day of dissection, the temporal bones were thawed at room temperature, placed in the surgical position, and subjected to mastoidectomy and posterior tympanotomy under a microscopic view. Following the identification of the RW niche, false membranes or mucous folds, when present, were removed to expose the RW membrane. In addition, any bony projections that restricted the visualization of the RW membrane were drilled, keeping the RW membrane intact. The quadrants of the round window were divided visually. Two perpendicular lines were drawn. The first was drawn at larger longitudinal axis of the round window membrane and was called line a. The second was drawn in the middle of line a and was called line b. These lines define the anterosuperior (I) and the anteroinferior quadrant (II) (Figure 1).

**Figure 2:**
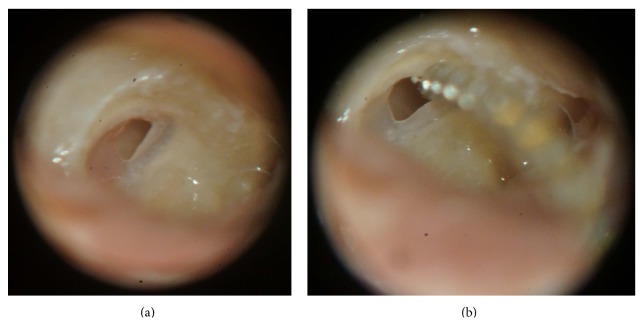
Incision and electrode array insertion through the anterosuperior quadrant of the round window membrane. (a) Incision in the anterosuperior quadrant of the round window membrane. (b) Beginning of the insertion of the electrode array through the opening in the anterosuperior quadrant.

**Figure 3:**
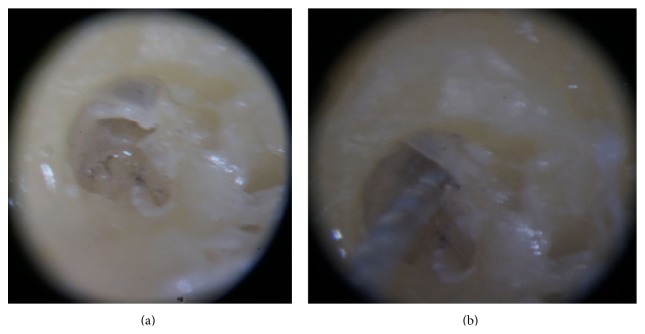
Incision and electrode array insertion through the anteroinferior quadrant of the round window membrane. (a) Incision in the anteroinferior quadrant of the round window membrane. (b) Beginning of the insertion of the electrode array through the opening in the anteroinferior quadrant.

The RW membrane was incised in the anterosuperior or anteroinferior quadrant (Figures 2 and 3). The point of insertion of the electrode array was always close to the annulus of the selected quadrant.

The electrode arrays were inserted using appropriate instruments through the openings in the respective quadrants by a single surgeon experienced in CI placement. The stapes footplate on all bones was removed to allow the flow of compounds used for histological examination throughout the cochlea. The electrode arrays were fixed with ethyl-cyanoacrylate glue in the region of the posterior tympanotomy.

The electrode array EVO (Oticon Medical, Gothenburg, Sweden; Oticon Medical/Neurelec, Vallauris, France), a straight array with a smooth surface and carrying 20 electrodes, was used. The total length was 24 mm, proximal diameter was 0.5 mm, and distal tip diameter was 0.4 mm [16].

After electrode array insertion, the bones were fixed in 10% formaldehyde and dehydrated using ethanol in increasing concentrations of 70%–100% and 100% acetone. The dehydrated bones were embedded in epoxy resin, placed in desiccators, and subjected to vacuum to promote resin penetration throughout the cochlea and to eliminate air bubbles. The histological examination procedure has been previously detailed by Plenk [17].

All the embedded bones were subjected to tomography to confirm the intracochlear position of the electrode array and to rule out kinking. Computed tomography was also performed to determine the position and orientation of the cochlea within the temporal bone block and to define the accurate plane for sectioning in each specimen.

Next, the epoxy blocks were transferred to a microgrinding machine, polished, and stained with toluidine blue. The stained surfaces were examined using a stereomicroscope under magnifications of 15x, 30x, 60x, 94x, and 120x and photographed. After image collection, the bone samples were polished once again to expose new surfaces; this procedure was repeated for every 500 *μ*m until the entire cochlea could be visualized. The microgrinding technique has been previously detailed by Stöver et al. [18].

Blinded individuals who were experienced and comfortable with cochlear histopathology performed histological sectioning and analyses.

**Figure 4:**
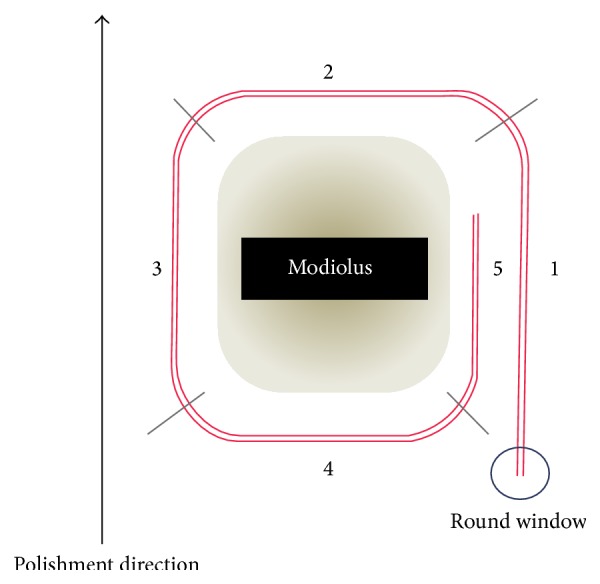
Diagrammatic representation of the intracochlear electrode array (red) and the division of the cochlea into five segments.

During histological analysis, each cochlea was divided into five segments to standardize the intracochlear regions (Figure 4).

The beginning of segment 1 corresponded to the RW membrane, while that of segments 2 and 4 indicated the surface where the modiolus was no longer visualized. In the latter two segments, the electrode could be visualized in the transverse orientation.

Intracochlear trauma observed by histological analysis was graded according to the classification proposed by Eshraghi et al. [19]: grade 0, no trauma; grade 1, elevation of the basilar membrane; grade 2, rupture of the basilar membrane; grade 3, dislocation of the electrode array to the scala vestibuli; and grade 4, severe trauma such as fracture of the osseous spiral lamina, modiolus, or stria vascularis.

Two separate analyses of intracochlear trauma were performed for each segment in each group. In the first analysis, any trauma beyond grade 0 was considered positive trauma. In the second analysis, as reported in previous studies [12], only grade 2, 3, or 4 trauma was considered positive trauma. Grade 1 trauma was analyzed together with grade 0 trauma because it represented only elevation of the basilar membrane.

The numbers of temporal bones available for research were limited, so convenience sample, with 25 temporal bones, was adopted. The data obtained in the study were entered into a Microsoft Excel spreadsheet. In both groups, the number of exposed surfaces per sample, the presence of intracochlear trauma, and degree of trauma in each segment have been described with the use of absolute and relative frequencies. The existence of association between the presence of intracochlear trauma and the quadrant of insertion of the electrode array was verified by exact test of Fisher. The degrees of trauma in each segment were compared between groups with the Mann-Whitney test. In all tests, the descriptive level (*P* value) ≤ 0.05 was considered statistically significant.

## 3. Results

No temporal bone was excluded. The electrode array was inserted through the anterosuperior and anteroinferior quadrants of the RW membrane in 13 and 12 samples, respectively.

In all samples, drilling of bony projections in the RW niche was required because they restricted visibility of and accessibility to the RW membrane. Complete insertion of the electrode array was possible in 24 samples with minimal or no resistance. In the remaining sample, the electrode array receded a few millimeters after insertion. There were no technical limitations to electrode placement in the randomized quadrant in any of the temporal bone samples.

**Figure 5:**
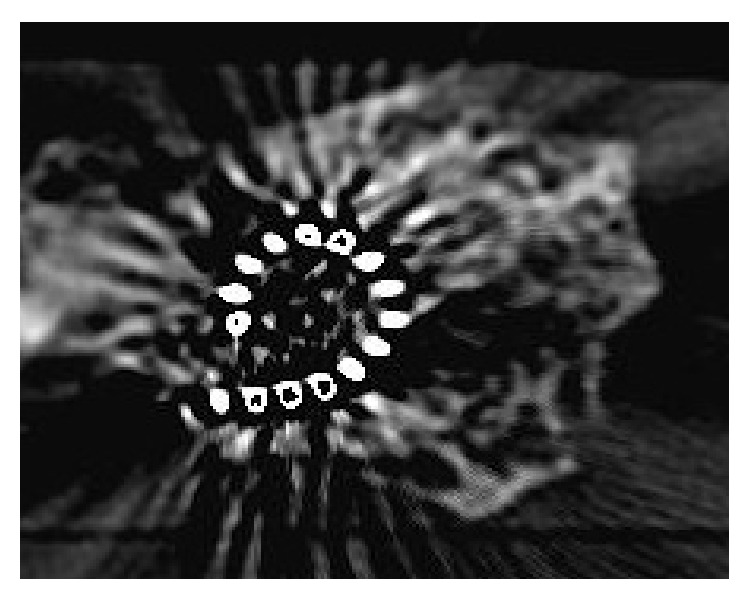
Computed tomography imaging with visualization of the electrode array within the cochlea.

All samples were subjected to computed tomography. The electrode arrays could be visualized inside the cochlea in all temporal bones. No kinking was observed in any array (Figure 5).

All bone surfaces from the RW membrane to the end of the cochlea were analyzed.

**Figure 6:**
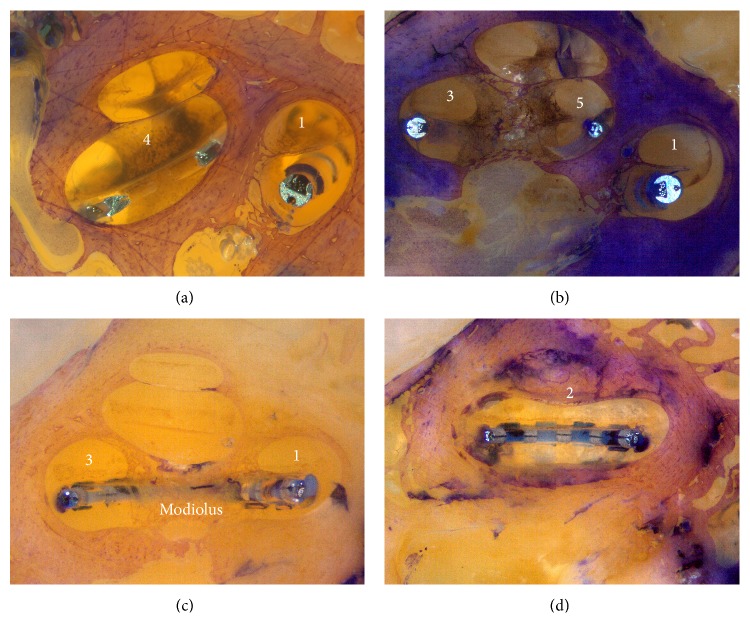
(a) Identification of segments 1 and 4. The modiolus is located deeper than the bone surface. (b) Identification of segments 1, 3, and 5. The modiolus is now visible on the surface of the bone. This defines the beginning of segments 3 and 5. (c) Visualization of segments 1 and 3 and the modiolus. (d) Visualization of the beginning of segment 2. The modiolus can no longer be visualized.

Figure 6 shows the different intracochlear segments. Figures 6(c) and 6(d) represent the same temporal bone.  Figure 6(c) shows the last surface where the modiolus could be visualized. In the next surface, shown in Figure 6(d), the modiolus could no longer be visualized, defining the beginning of segment 2.

Computed tomography helped in determining the orientation of the cochlea within the temporal bone block and in defining the accurate plane to initiate polishing in each specimen.

A total of 372 bone surfaces were obtained for histological analysis. None of these surfaces exhibited artifacts that could prevent identification of the intracochlear structures.

Intracochlear trauma, when present, was clearly visible and could be graded according to the classification proposed by Eshraghi et al. [19].

**Figure 7:**
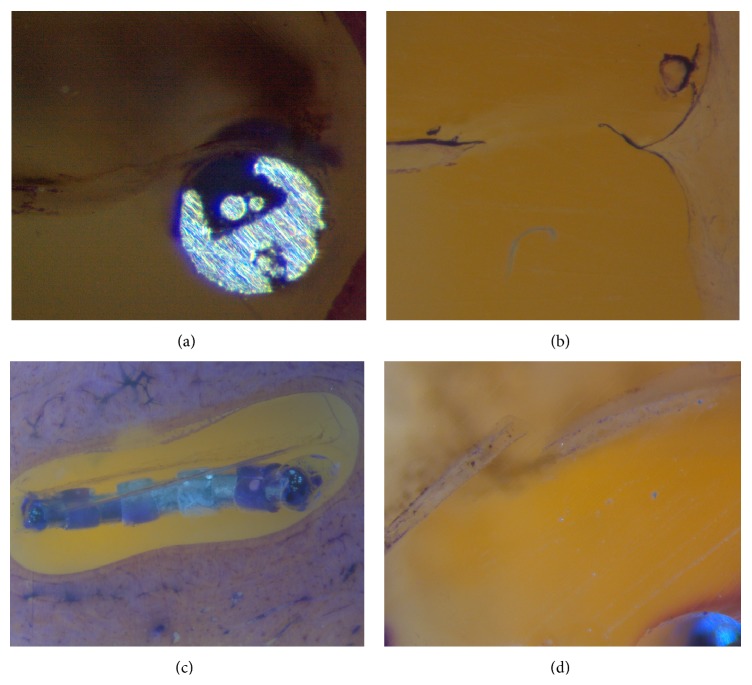
(a) Electrode array in the scala tympani causing slight elevation of the basilar membrane (grade 1). (b) Identification of a ruptured basilar membrane (grade 2). (c) Dislocation of the electrode array from the scala tympani to the scala vestibuli (grade 3). (d) Identification of a fracture in the osseous spiral lamina (grade 4).

Distinct damage to the intracochlear structures was identified. In all bones, the damage did not extend beyond the end of the CI electrode tip. When the same segment exhibited multiple grades of trauma, the trauma with the highest grade was considered.  Figure 7 shows examples of histological surfaces, which illustrate the classification system for intracochlear trauma proposed by Eshraghi et al. [19].

The presence of intracochlear trauma in all segments according to both above-mentioned criteria (considering any trauma beyond grade 0 as positive trauma or considering grade 2, 3, or 4 trauma as positive trauma) showed no significant correlation with the quadrant of insertion (*P* > 0.05).

Furthermore, there were no significant differences in the grade of intracochlear trauma in each segment between the two groups (*P* > 0.05; Table 1).

## 4. Discussion

The present study demonstrated a similar degree of intracochlear trauma when CI electrode arrays were inserted through the anterosuperior and anteroinferior quadrants of the RW membrane.

The insertion of electrode arrays is considered an essential step of atraumatic surgery. Numerous authors have compared the advantages and disadvantages of CI insertion through cochleostomies performed in different positions. In this regard, cochleostomies performed at the inferior margin of RW are considered less traumatic and are associated with a lower frequency of erroneous electrode array placement in the scala media or scala vestibuli and a higher probability of residual hearing preservation compared with cochleostomies performed in the superior, anterior, and anteroinferior regions of the RW [20–24]. On the basis of these studies identifying differences in intracochlear trauma and audiological performance after CI electrode array insertion through different cochleostomy locations, our research group evaluated differences in intracochlear trauma caused by electrode array insertion through different quadrants of the RW membrane.

In the present study, the electrode arrays were inserted through the anterior segment of the RW membrane on the basis of the following findings in anatomical studies: difficulty in visualization of the posterior segment of the RW membrane because of its horizontal orientation, close proximity of the posterior segment to the inner ear structures, and the possibility of injuries to the osseous spiral lamina during drilling of the posterior bony projections for adequate visualization of the posterior margin of the RW membrane [3, 21, 23]. Therefore, we opted to compare the anterior quadrants of the RW membrane (anterosuperior and anteroinferior) and for no mandatory viewing of the posterior segment of the RW membrane.

Roland et al. [21] studied 15 temporal bones and estimated that the area covered by the vertical or anterior portion of the RW membrane varies from 0.8 to 1.75 mm^2^, with a mean of 1.39 mm^2^. We calculated a 0.19 mm^2^ area occupied by the larger diameter of the electrode array using the following formula: *A* = *π* · *r* ^2^. Mathematically, this facilitates insertion of the electrode array through any of the anterior quadrants of the RW membrane.

During the surgeries performed in the present study, bones overhangs of the RW niche that limited visualization of the membrane were observed in all samples, necessitating drilling of the anterior and anteroinferior bony projections. When necessary, the posterior projections were minimally drilled to improve exposure of the anterior margin and facilitate electrode array insertion. These results are in agreement with those of previous anatomical studies [5, 24], particularly the study by Roland et al. [21], who dissected 30 temporal bones and observed that anatomical projections restricted the visualization of the RW membrane in all samples. Furthermore, the need for drilling the promontory could not be ruled out in any of the samples, although the amount of drilling required would be minimal. According to Takahashi and Sando [25], electrode array insertion through the RW membrane required the removal of <1 mm of bone, which is lesser than that required for cochleostomy and decreases the possibility of trauma [14, 26].

The statistical analysis in the present study was two-tailed, considering that previous data did not indicate the direction in which the results were statistically significant. Indeed, the literature on this issue is conflicting. The hypothesis that intracochlear trauma is lesser with electrode array insertion through the anteroinferior quadrant of the RW membrane was raised because of the proximity of this quadrant to the inferior margin of RW, and, to date, cochleostomy at this margin has been considered less traumatic [20]. On the other hand, because of the downward direction of the scala tympani from RW, there is a higher probability of the electrode arrays being directed towards the superior structures of the scala tympani during insertion through the anteroinferior quadrant [22, 27]. This suggests that the probability of intracochlear trauma is lower when the arrays are inserted through the anterosuperior quadrant of the RW membrane.

Briggs et al. [20] reported that cochleostomy at the inferior margin of RW requires complete skeletonization of the facial nerve and chorda tympani; therefore, many surgeons avoid cochleostomy at this location. In this study, we could visualize the anterior segment of the RW membrane in all bone samples. However, to expose the anteroinferior quadrant, enlargement of the posterior tympanotomy was necessary, with skeletonization of the mastoid segment of the facial nerve in some samples. Skeletonization would not be necessary to visualize only the anterosuperior quadrant; this observation is in agreement with that in previous studies. Although these studies did not involve electrode array insertion through RW, the surgical exposure required for cochleostomy at the inferior margin of RW is very similar to that required for insertion through the anteroinferior quadrant of the RW membrane. According to the results of the present study, surgeons who avoid more extensive dissection of the facial nerve for visualization of the anteroinferior quadrant of the RW membrane can implant electrode arrays through the anterosuperior quadrant, which requires less exposure.

Currently, the microgrinding technique is the most powerful technique for determining the localization of electrodes and insertion trauma to the cochlea [18]. The use of nondecalcified human temporal bones permits the* in situ* evaluation of intracochlear trauma caused by CI placement [28]. The primary advantages of this method are that the excellent image quality enables clear identification of the intracochlear structures, the electrode array, and the relationship between these two components. However, the microgrinding technique has some limitations. The cut sections are not preserved, and therefore evaluation of the temporal bone is limited to the time during which sectioning is performed and depends on the quality of photographic documentation. Another limitation is the time-consuming and cost-intensive method of electrode evaluation. Despite the disadvantages, this technique provides data of, so far, unknown clarity regarding the detection and localization of insertion trauma [18].

Assessment of the insertion depths of the electrode arrays was beyond the scope of this study; therefore, we decided to standardize the segmentation of the cochlea in a more visual manner, rather than segmentation in degrees, which could be achieved under computed tomography guidance.

To standardize the histological analysis was opted for classification system proposed by Eshraghi et al. [19] in 2003. This is the most widely used classification in histological studies of intracochlear trauma. However, it is considered a disadvantage of this classification that the negative functional consequences generated by an intracochlear trauma do not evolve according to the ascending numbering classification. As an example, consider a trauma grade 4, because of a fracture in the osseous spiral lamina. Although this trauma changes the cochlear function in the affected region, probably it does not interfere with gradients of ions and hemodynamics of intracochlear liquids. On the other hand, a level 2 trauma, caused by rupture of the basilar membrane, presumably leads to more dispersed intracochlear damages. This occurs because this type of trauma causes the mixture of endolymph and perilymph, altering the normal gradient of the ions, which can lead to degeneration of hair cells and neural structures. Because of endocochlear flow all the cochlea may be compromised [29, 30].

This study has some limitations. First, dynamic monitoring of the electrode array during insertion was not implemented; therefore, we cannot describe if the trajectory differed with insertion through different quadrants of the RW membrane. Second, our study was a small study comparing two variables, and further larger studies to clarify our findings are required. Third, we used only straight electrodes; therefore, our results are not applicable to precurved electrodes.

## 5. Conclusions

In conclusion, there were no significant differences in the incidence and severity of intracochlear trauma caused by insertion of electrode arrays through the anterosuperior and anteroinferior quadrants of the RW membrane. Preservation of the fine intracochlear structures continues to be an important topic.

## Figures and Tables

**Table 1:** Grade of intracochlear trauma caused by electrode array insertion through the anterosuperior or anteroinferior quadrant of the round window membrane in each segment.

	Insertion				Total		
Variable	Group 1 (*N* = 13)		Group 2 (*N* = 12)		(*N* = 25)		*P* ^§^
	*N*	%	*N*	%	*N*	%	
Segment 1 (grade)							0.503
0	13	100	10	83.3	23	92.0	
1	0	0	0	0	0	0	
2	0	0	0	0	0	0	
3	0	0	0	0	0	0	
4	0	0	2	16.7	2	8.0	
Segment 2 (grade)							0.538
0	10	76.9	8	66.7	18	72.0	
1	1	7.7	0	0	1	4.0	
2	1	7.7	1	8.3	2	8.0	
3	1	7.7	1	8.3	2	8.0	
4	0	0	2	16.7	2	8.0	
Segment 3 (grade)							0.470
0	9	69.2	7	58.3	16	64.0	
1	2	15.4	0	0	2	8.0	
2	1	7.7	2	16.7	3	12.0	
3	0	0	0	0	0	0	
4	1	7.7	3	25.0	4	16.0	
Segment 4 (grade)							0.894
0	11	84.6	10	83.3	21	84.0	
1	1	7.7	0	0	1	4.0	
2	0	0	0	0	0	0	
3	1	7.7	1	8.3	2	8.0	
4	0	0	1	8.3	1	4.0	
Segment 5 (grade)							0.810
0	10	76.9	9	75.0	19	76.0	
1	1	7.7	0	0	1	4.0	
2	0	0	0	0	0	0	
3	1	7.7	0	0	1	4.0	
4	1	7.7	3	25.0	4	16.0	
Lowest grade							0.406
0	9	69.2	7	58.3	16	64.0	
1	1	7.7	0	0	1	4.0	
2	1	7.7	0	0	1	4.0	
3	1	7.7	0	0	1	4.0	
4	1	7.7	5	41.7	6	24.0	

Group 1: insertion through the anterosuperior quadrant of the round window membrane; group 2: insertion through the anteroinferior quadrant.

*N* = number of samples.

^§^Results of the Mann-Whitney *U* test.
